# Impact of Endurance and Resistance Training on Skeletal Muscle Glucose Metabolism in Older Adults

**DOI:** 10.3390/nu11112636

**Published:** 2019-11-03

**Authors:** Leslie A. Consitt, Courtney Dudley, Gunjan Saxena

**Affiliations:** 1 Department of Biomedical Sciences, Ohio University, Athens, OH 45701, USA; saxenag@ohio.edu; 2 Ohio Musculoskeletal and Neurological Institute, Ohio University, Athens, OH 45701, USA; 3 Diabetes Institute, Ohio University, Athens, OH 45701, USA; 4 Department of Biological Sciences, Ohio University, Athens, OH 45701, USA; cd630016@ohio.edu

**Keywords:** age, skeletal muscle, insulin sensitivity, endurance exercise, resistance exercise, AS160, pyruvate dehydrogenase, glycogen

## Abstract

Aging is associated with insulin resistance and the development of type 2 diabetes. While this process is multifaceted, age-related changes to skeletal muscle are expected to contribute to impaired glucose metabolism. Some of these changes include sarcopenia, impaired insulin signaling, and imbalances in glucose utilization. Endurance and resistance exercise training have been endorsed as interventions to improve glucose tolerance and whole-body insulin sensitivity in the elderly. While both types of exercise generally increase insulin sensitivity in older adults, the metabolic pathways through which this occurs can differ and can be dependent on preexisting conditions including obesity and type 2 diabetes. In this review, we will first highlight age-related changes to skeletal muscle which can contribute to insulin resistance, followed by a comparison of endurance and resistance training adaptations to insulin-stimulated glucose metabolism in older adults.

## 1. Age and Insulin Resistance

It is estimated that 30% of individuals over the age of 60 are affected by type 2 diabetes [1]. Aging has been associated with glucose intolerance and whole-body insulin resistance [2,3,4,5,6,7,8], with the factors contributing to these disorders being complex and likely multifaceted including chronological age [2], reduced physical activity [9], inflammation [10], and/or increased body fat [9,11]. Since skeletal muscle is the primary target for insulin-mediated glucose uptake [12], age-related changes in the structure and metabolism of this tissue are also thought to play a major role in the pathogenesis of insulin resistance in older adults.

## 2. Age and Skeletal Muscle Atrophy

Age-associated muscle atrophy begins as early as 25 years of age and accelerates thereafter, so that, by 80 years of age, approximately 40% of the vastus lateralis (muscle in the thigh) has been lost [13]. Much of the current literature discussing age-related loss of muscle mass (termed sarcopenia) focuses on the adverse effects to muscular strength and power, leading to loss of mobility and the inability to perform daily activities, including climbing stairs and lifting objects [14]. While these are obviously critical concerns, it should also be noted that sarcopenia is thought to have harmful effects on glucose uptake, since it reduces the amount of available muscle mass for insulin-stimulated glucose disposal [15].

Early work by Lexell et al. [13] reported that the atrophy that takes place with aging is due to a loss of both type I (oxidative) and type II (glycolytic) muscle fibers and a reduction in fiber size (cross-sectional area) that primarily affects type II fibers [13]. These findings were later supported by Coggan and co-workers (1992) [16] who reported that the cross-sectional area of type IIa and IIb in the gastrocnemius was reduced in aged individuals compared to their younger counterparts, despite no differences in the percentage of type I and type II fibers.

In addition to the loss of cross-sectional area, type II fibers also appear to be negatively targeted by the aging process with respect to glucose metabolism. Single fiber proteomic analysis revealed that proteins involved in glycolysis and glycogen metabolism were downregulated in type II fibers of regularly active aged, compared to young individuals [17]. Additionally, GLUT4 protein, the transporter responsible for insulin-stimulated glucose uptake, has been reported to be reduced in type II, but not type I fibers, in older (~64 years) compared younger (~29 years) adults [18]. While sarcopenia likely plays a role in insulin resistance, the age-related metabolic and cellular changes that occur within skeletal muscle are thought to have a prominent role and have been the focus of researchers attempting to elucidate the intracellular mechanisms responsible for age-related insulin resistance. The current review will outline several of these mechanisms, followed by a discussion and comparison of two effective interventions for improving insulin sensitivity in the elderly: endurance and resistance exercise training.

## 3. Age and Skeletal Muscle Insulin Resistance

**Figure 1:**
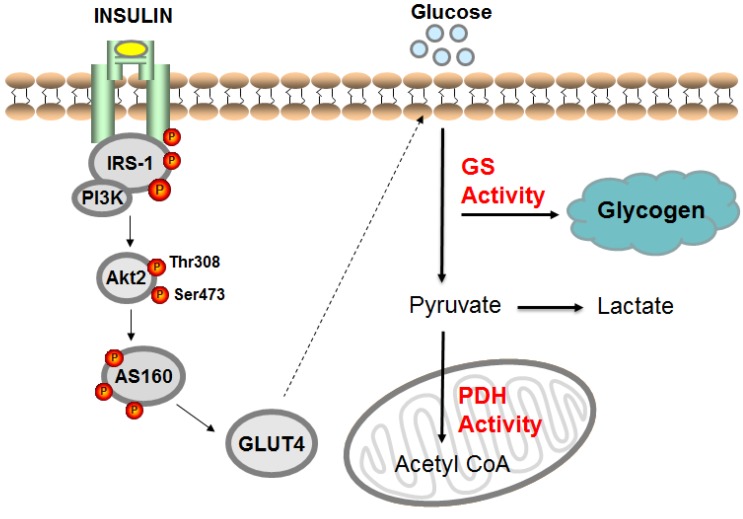
Overview of insulin-stimulated skeletal muscle glucose metabolism in humans. Once insulin binds to the insulin receptor, it activates a signaling cascade resulting in glucose transporter type 4 (GLUT4) translocation to the plasma membrane which facilitates glucose uptake into the muscle cell. The majority of glucose that enters the cell is either stored as glycogen, oxidized in the mitochondria, or converted to lactate. IRS-1: insulin receptor substrate 1; PI3K: phosphoinositide 3-kinase; GS: glycogen synthase; PDH: pyruvate dehydrogenase.

To investigate the cellular mechanisms responsible for reduced insulin-stimulated glucose uptake, several studies have examined the insulin signaling cascade in skeletal muscle. Age-related impairments in skeletal muscle insulin signaling have been reported in both human [2,8] and animal [19,20,21] models. In healthy, insulin sensitive individuals, insulin binds to the insulin receptor to initiate the signaling cascade which involves phosphorylation of the insulin receptor, insulin receptor substrate-1 (IRS-1) association with phosphoinositide 3-kinase (PI3K), Akt2 phosphorylation on threonine 308 and serine 473 sites, and AS160 phosphorylation on numerous sites, allowing the translocation of glucose transporter type 4 (GLUT4) to the plasma membrane to facilitate glucose uptake [22,23]. After glucose enters the myocyte (muscle cell), it undergoes either nonoxidative glucose disposal (primarily glycogen synthesis) or mitochondrial glucose oxidation (Figure 1).

To investigate if impairments in insulin signaling could contribute to age-related insulin resistance, our group studied sedentary men and women across a wide range of adult life span (18–84 years) with biopsies of the vastus lateralis at baseline and 60 min of a hyperinsulinemic-euglycemic clamp [2]. We determined that insulin-stimulated phosphorylation of AS160 on serine-588, threonine-642, and serine-666 (but not serine-318 or 751) were impaired in conjunction with the whole-body insulin resistance observed with advancing age [2]. Previous research has determined that insulin induces the phosphorylation of AS160 on serine-318, serine-588, serine-751 and threonine-642 [24], and that these sites lie within the consensus sequence for Akt phosphorylation [24]. Of interest, insulin-stimulated phosphorylation of Akt on serine-473 was retained in our older adults, suggesting another kinase and/or phosphatase known to regulate AS160 phosphorylation [25,26,27] plays a role in age-related impairments in AS160 phosphorylation. Regardless of the source of impairment, the fact that aging had a negative effect on insulin-stimulated serine-588 and threonine-642 phosphorylation is of critical significance given that these two sites are believed to have the greatest combined impact on GLUT4 translocation [24].

In contrast to our findings, Peterson et al. [8], reported impairments upstream of AS160 in aged individuals that were matched to younger individuals for body mass index (BMI), fat mass, and habitual physical activity. In that study, older adults had reduced skeletal muscle Akt activity after 20 min of hyperinsulinemia, compared to their younger counterparts. It is possible that the different findings are due to measuring phosphorylation versus activity; however, it is also conceivable that the timing of the Akt measurement played a role. We have previously reported that older adults have a delayed metabolic response to hyperinsulinemia [28] and at 20 min of insulin stimulation, neither young or old individuals would have reached steady state glucose uptake. Therefore, it is possible that in older adults, skeletal muscle Akt activity is blunted early during hyperinsulinemia, but normalizes to their younger counterparts when steady state is achieved; however, age-related defects in site-specific AS160 phosphorylation remain, contributing at least in part to insulin resistance in older adults. To date, no known studies have examined the effects of age on insulin signaling in a fiber-type manner in humans; however, insulin-stimulated Akt phosphorylation in isolated rat soleus (primarily type I fibers), but not epitrochlearis (primarily type II fibers) has been reported to be impaired in aged rats [20], suggesting future studies should be conducted in humans.

Skeletal muscle GLUT4 has been reported to be reduced with aging in some [2,18,29], but not all [29,30,31] studies. It is possible that these inconsistent findings are due in part to fiber-type variability between subjects. Gaster et al. [18] reported that older (mean age: 64 years) individuals had ~25% less GLUT4 in type II fibers of the vastus lateralis compared to young (mean age: 29 years) subjects, whereas no differences were observed in type I fibers. While the amount of GLUT4 content in the whole muscle is important, the amount of GLUT4 specifically located in the plasma membrane in response insulin-stimulated signaling is of greater physiological relevance. Unfortunately, due to the amount of tissue required for plasma membrane isolation by traditional techniques, no known studies have investigated the effects of human aging on GLUT4 trafficking. It has been reported that in the quadriceps of old (24 month) rats, the ability to translocate GLUT4 to the plasma membrane in response to insulin is impaired [32]. Taken together, these results suggest that age-related impairments in insulin signaling likely contribute to reduced trafficking of GLUT4 from intracellular GLUT4 storage vesicles (GSVs) to the plasma membrane, ultimately contributing to insulin resistance with aging.

## 4. Age and Nonoxidative (Glycogen Synthesis) and Oxidative Pathways

Insulin-stimulated nonoxidative disposal rates have been reported to be diminished in elderly individuals compared to their younger counterparts [33,34,35]. While nonoxidative metabolism can include pathways other than glycogen synthesis (i.e., the pentose phosphate and hexosamine biosynthetic pathway), most skeletal muscle nonoxidative metabolism occurs through the glycogen synthesis pathway, and this will be the focus of the current review. Early research demonstrated that resting skeletal muscle glycogen stores were ~60% lower in old compared to young individuals [36], suggesting age-related impairments in the glycogen synthase pathway. In support of this, insulin-stimulated skeletal muscle glycogen synthase activity has been reported to be diminished in older adults [34,37] when compared to the response of younger individuals [34,38].

There is also evidence supporting impairments in insulin-stimulated glucose oxidation in the elderly when matching for weight and BMI [39], and when normalizing oxidation rates to total glucose disposal [40]. The fact that older adults have reduced insulin-stimulated glucose uptake (likely via reduced insulin signaling and possibly GLUT4 as discussed above) often confounds the ability to accurately compare intracellular pathways responsible for glucose utilization within the muscle. For example, reduced activity of an intracellular pathway (either oxidation or glycogen synthase) in older adults may simply be due to decreased glucose entering the muscle (i.e., decreased glucose availability). To control for the reduced glucose uptake in older adults, Gumbiner et al. [40] matched insulin-stimulated glucose uptake in older individuals to that of their younger counterparts and reported that whole-body glucose oxidation was diminished in the aged individuals, whereas nonoxidative metabolism including measurements of skeletal muscle glycogen synthase was similar between the groups. Based on their findings, they concluded that age-related reductions in insulin-stimulated glucose oxidation contribute to the insulin resistance associated with aging, independent of the defect of glucose uptake.

Our group has speculated that intramuscular glucose may be preferentially shuttled away from oxidation towards anaerobic metabolism in older adults when controlling for insulin-stimulated glucose uptake. During a hyperinsulinemic-euglycemic clamp, we reported that aged individuals had higher plasma lactate levels than their younger counterparts after normalizing to glucose uptake. Of particular interest, insulin-stimulated increases in lactate were associated with impaired skeletal muscle pyruvate dehydrogenase (PDH) regulation (phosphorylation) [41]. PDH is a mitochondrial enzyme that allows for the by-product of glucose (pyruvate) to be converted to acetyl-CoA and enter the citric acid cycle for oxidation. Reduced skeletal muscle PDH flux in the elderly during insulin stimulation has also been reported by others [8], highlighting a potential mechanism contributing to diminished glucose oxidation. Taken together, these studies provide evidence that during insulin-stimulated conditions, skeletal muscle PDH regulation may be compromised with aging, resulting in reduced glucose oxidation, which could have detrimental effects on metabolic flexibility and insulin sensitivity [42,43].

## 5. Physical Activity, Exercise, and Insulin Sensitivity in Older Adults

Physical activity has long been associated with superior health and enhanced quality of life in individuals of advancing age [44]. Research from the Harvard Alumni Health Study suggested that older men (mean age: 66 years) without a major health risk could reduce their risk of dying by becoming a ‘weekend warrior’ (at least 1000 kcal/week) [45], suggesting that even one to two exercise sessions a week can prolong a person’s lifespan. Older adults who have been physically active throughout their lifetime have been found to have superior levels of metabolic health, compared to inactive older adults [46]. Many researchers contend that declining physical activity with aging is a main contributing factor in determining the degree of age-related insulin resistance [9]. Physical inactivity has been reported to be responsible for 7% of type 2 diabetes cases [47] and that inclusion of physical activity can increase an individual’s lifetime spent without diabetes [48]. Whereas, traditional research has been focused on trying to determine exercise intensity requirements for improved insulin sensitivity, current research suggests that total accumulated activity time is the most important variable to improve insulin sensitivity in previously sedentary individuals [49]. Given the importance of skeletal muscle in glucose disposal, it is likely the muscular adaptations that take place in response to exercise-induced muscle contractions contribute to the improved insulin sensitivity. While not the focus of the current paper, the effects of acute exercise on metabolism should not be negated, and is reviewed elsewhere [50]. A number of controlled exercise training studies have demonstrated improved whole-body insulin sensitivity and skeletal muscle metabolism in older adults, comparable to that of their younger counterparts, making it an effective intervention to improve or prevent insulin resistance in the aging population [2,3,51,52,53]. The following will describe the documented skeletal muscle intracellular adaptations that contribute to improved insulin sensitivity in older adults in response to either endurance or resistance training.

## 6. Endurance Training

Early research examining the effects of endurance training on middle- to older-aged men demonstrated that they were capable of comparable improvements in whole-body aerobic capacity and skeletal muscle metabolic adaptations as their younger counterparts [54]. Subsequent studies have aimed to examine the cellular mechanism(s) in skeletal muscle through which endurance training may improve insulin sensitivity in older adults, including improvements in the insulin signaling cascade, and glucose utilization.

### 6.1. Endurance Training and Skeletal Muscle Insulin Signaling in Older Adults

Despite reports that endurance training increases skeletal muscle Akt protein content in aged individuals [37,55], it does not appear that insulin-stimulated Akt phosphorylation is changed when normalized to total protein in older [2,55] or younger individuals [56]. While these findings imply Akt capacity may be increased due to increased protein, they also suggest that if improved sensitivity/efficiency is present, it is located downstream of Akt. In support of this, our group reported that 3 months of moderate-intensity (70–75% VO2peak) endurance training increased insulin-stimulated AS160 phosphorylation on serine site 588 (~25%) in both young and aged, non-obese men and women [2] when normalized to AS160 total protein. Of particular interest, we also observed improvements in insulin-stimulated AS160 threonine-642 (~57%) and serine-666 (~80%) phosphorylation sites in aged, but not young individuals, suggesting older individuals may be particularly responsive to moderate-intensity endurance training with respect to improved AS160 phosphorylation [2]. While the effects of AS160 serine-666 on GLUT4 translocation remain unclear, these findings support a mechanism of improved insulin sensitivity since AS160 threonine-642 is critical for glucose uptake and the enhanced phosphorylation on serine-588 would have additional stimulatory effects on GLUT4 translocation [24].

Preliminary evidence suggests increased body fat in both old and young individuals may diminish the effects of exercise training on AS160. In contrast to our results in non-obese individuals, 6 months of moderate-intensity (60–70% VO2peak) endurance training in obese, older adults that included diet-induced weight loss, did not improve insulin-stimulated AS160 phosphorylation (using antibodies designed to preferentially detect site threonine 642) [57]. Similarly, short-term training in young, obese individuals was found to have no effect on insulin-stimulated AS160 phosphorylation [58].

High-intensity exercise training has received considerable attention over the past few years given its reduced time commitment and proven ability to increase insulin sensitivity in young individuals [59]. Data suggest that these insulin sensitizing effects can be extended to older individuals [52,60]. Moreover, it has been reported that in the absence of weight loss, high-intensity, not moderate-intensity exercise, is required for improvements in whole-body insulin sensitivity in obese, elderly individuals [52]. Recently, Mandrup et al. [60] investigated the effects of 3 months of high-intensity aerobic exercise training (cycling) in sedentary, normal-weight, postmenopausal women. Results indicated that high-intensity exercise increased insulin-stimulated glucose uptake and skeletal muscle Akt phosphorylation (but not glycogen synthase activity), differing from previous findings involving moderate-intensity exercise (discussed above and below). While this is the only known study investigating the effects of high-intensity exercise training on insulin signaling in older subjects, it provides evidence of improved insulin action in muscle, possibly via a different cellular mechanism than traditional moderate-intensity endurance training. Further research examining the effects of high-intensity exercise training downstream of Akt are warranted, especially in obese, older adults.

In addition to enhanced insulin signaling, increased GLUT4 availability could play a role in the improvements in insulin sensitivity with endurance training in elderly individuals. Moderate-intensity endurance training ranging from seven days to six months has been reported to increase skeletal muscle GLUT4 protein content between 10% and 106% in aged individuals [2,30,31,37,55,61,62]. Of interest, the shortest time period of training had the greatest gains in GLUT4. Cox et al. [30] reported that seven consecutive days of cycling for 1 h at ~75% VO2peak increased skeletal muscle GLUT4 in older women (54%) and men (106%), with similar increases observed in their younger counterparts. These data, along with results in young individuals [63], suggest that moderate-intensity, high-frequency, endurance training significantly increases GLUT4 within a week, especially if that muscle is heavily recruited (i.e., vastus during cycling) with no additional improvements observed with subsequent weeks of training. Unlike improvements in insulin signaling that may be compromised with obesity, training-induced increases in GLUT4 have been reported in older, obese individuals [55], as well as older men with type 2 diabetes [31].

In summary, it appears that improvements in the insulin signaling cascade and increased GLUT4 availability may contribute, at least in part, to enhanced insulin sensitivity in older, non-obese individuals in response to moderate- to high-intensity endurance training. Older adults that are obese and/or type 2 diabetic can receive the benefits of increased GLUT4 protein content with endurance training, which may contribute to their improved insulin sensitivity with this type of exercise training.

### 6.2. Endurance Training and the Glycogen Synthase Pathway in Older Adults

Exercise-induced improvements in insulin sensitivity have also been linked to enhancements in metabolic pathways responsible for glucose utilization. Previous research [52] reported that 12 weeks of high-intensity exercise training in overweight, elderly (mean age: 74 years) individuals increased insulin sensitivity, via nonoxidative glucose disposal, suggesting improvements in the skeletal muscle glycogen synthesis pathway. In support of this, older adults have been reported to increase skeletal muscle glycogen content with endurance training [36,60,62,64], with at least one study reporting they are more responsive to exercise-induced glycogen accumulation than their younger counterparts [36]. Based on these findings, it is not surprising that enhancements in the glycogen synthase pathway have been observed in elderly individuals in response to endurance training [37]. Bienso et al. [37] reported that 8 weeks of endurance training in healthy, lean, elderly men enhanced the glycogen synthase pathway, whereas PDH activity (responsible for glucose oxidation) was reduced in response to glucose ingestion. While it is conceivable to conclude from this study that endurance training evokes the preferentially shuttling of glucose towards storage over oxidation, the more physiological design of this study (consumption of glucose), resulted in a varying insulin response between individuals that may have influenced skeletal muscle signaling and nutritional demands. Regardless, this study demonstrates that improvements in insulin sensitivity (measured by the Matsuda Index) with endurance training paralleled enhancements in the glycogen synthase pathway in healthy, lean elderly men.

Whereas, training-induced enhancements in insulin signaling may be compromised in older, obese individuals, improvements in the glycogen synthase pathway appear to be obtainable in these individuals. Ryan et al. [57] reported that aged, overweight and obese men who underwent a 6-month program of endurance exercise training combined with diet-induced weight loss increased skeletal muscle fractional glycogen synthase activity. Moreover, whole-body insulin sensitivity post-intervention was related to improvements in insulin-induced glycogen synthase activity, independent of phosphorylation of Akt serine-473 or AS160 threonine-642. Similar improvements in insulin sensitivity and insulin-stimulated glycogen synthase activity were reported in obese, postmenopausal women undergoing an identical exercise and dietary intervention [65]. Of interest, this study [65] reported that improvements in insulin-stimulated glycogen synthase activity were stronger in women classified as glucose intolerant compared to those with normal glucose tolerance. Despite the inclusion of a dietary intervention in these previous studies [57,65], it does not appear that diet-induced weight loss is necessary to elicit improvements in the glycogen synthase pathway since 6 months of endurance training alone has been shown to increase insulin-stimulated glycogen synthase fractional activity [66]. Further, calorie restriction alone has been shown to have no effect on the glycogen synthase pathway in obese, older women [57]. Taken together, these findings suggest that older adults that are overweight, obese, and/or glucose intolerant, who may not benefit from improved Akt or AS160 signaling, can still receive the insulin sensitizing benefits of endurance training, possibly due to improvements in the skeletal muscle glycogen synthesis pathway.

### 6.3. Endurance Training and Glucose Oxidation Pathway in Older Adults

Several studies have reported mitochondrial deficits and reduced oxidative capacity in older adults [67,68,69]. Endurance exercise has long been established as an intervention to induce mitochondrial biogenesis in young individuals [70]. Early research demonstrated that elderly individuals may be more sensitive to exercise-induced increases in skeletal muscle oxidative capacity than their younger counterparts. For example, Meredith et al. [36] reported that 12 weeks of moderate-intensity endurance training was able to reverse age-related deficits in skeletal muscle oxidative capacity (~120%) in elderly individuals, whereas no changes were observed in younger individuals. Current research indicates increases in skeletal muscle mitochondrial biogenesis, and dynamic mitochondrial fission and fusion occur in response to endurance training, regardless of age [71]. Exercise-induced increases in skeletal muscle peroxisome proliferator-activated receptor-gamma coactivator (PGC)−1 alpha, considered the master regulator of mitochondrial biogenesis, occurs in both young and old individuals [72], and likely contributes to the increased mitochondrial content associated with endurance training.

The mitochondrial enzyme PDH is of interest due to its critical function in glucose oxidation and the fact that insulin-stimulated regulation has been reported to be diminished with age [8,41]. Of importance, 12 weeks of endurance training rescued age-related impairments in insulin-stimulated PDH regulation in older adults, which paralleled an improved (reduced) lactate response [41]. The conflicting PDH results between our study [41] and the work by Bienso et al. [37] may be related to the fact that PDH was measured in response to a hyperinsulinemic-euglycemic clamp in our study versus an oral glucose load in the Bienso et al. [37] study and/or that the former includes data from both sexes while the latter focused on the response in males only. Collectively, these results suggest that muscle plasticity with respect to the effects of endurance training on mitochondrial biogenesis is preserved with aging, allowing for improved skeletal muscle oxidative capacity and enhanced insulin-stimulated PDH regulation.

### 6.4. Endurance Training and Intramuscular Lipid (IMCL) in Older Adults

Increased intramuscular lipid (IMCL) has been reported in elderly individuals [4,73] and has been associated with insulin resistance [4,73,74]. Interestingly, intramuscular triglycerides (IMTG) have been reported to increase along with insulin sensitivity during endurance training [64,75,76,77,78]. Goodpaster et al. [75] was first to discover the athlete’s paradox, where young endurance-trained individuals were insulin sensitive despite having elevated IMTG stores equivalent to obese, insulin-resistant, type 2 diabetics. It is thought that the increased energy demands of endurance athletes, along with the increased mitochondrial content and function, allows for a higher rate of IMTG turnover (increased depletion/repletion cycles) that protects them from insulin resistance [75,79,80]. Of interest, this paradox is also observed in older individuals, where endurance training increases IMCL along with insulin sensitivity [64,76,77,78]. It is reasonable to assume that since older adults retain plasticity for exercise-induced mitochondrial biogenesis and oxidation, as discussed above, these adaptations account for the increases in IMCL in older adults, similar to their younger counterparts.

Subsequent to the athlete’s paradox findings, research has put less emphasis on IMTG as a direct cause of insulin resistance, and instead focused on lipid intermediates including diacylglycerol (DAG) and ceramide that have been directly linked to skeletal muscle insulin resistance [81] and are elevated in older adults [82]. Importantly, 16 weeks of endurance training in aged (60–75 years), obese individuals was reported to decrease skeletal muscle DAG (~−40%) and ceramide (~−30%), with exercise-induced improvements in insulin sensitivity associated with the decline in ceramides [64,78].

In summary, older adults, like their younger counterparts, experience the athlete’s paradox, where endurance training enhances insulin sensitivity in conjunction with increased IMTG stores. Perhaps of greater interest, endurance training in older adults has been found to be responsible for reducing IMCL species known to inhibit insulin signaling and insulin sensitivity, including ceramide and DAG.

## 7. Resistance Training and Skeletal Muscle Insulin Signaling in Older Adults

The effects of resistance training on glucose metabolism in older adults has unfortunately received less attention than endurance training; however, the American College of Sports Medicine recommends resistance training as a safe and effective lifestyle intervention for the elderly [83]. Like endurance training, resistance training has been linked to improved whole-body insulin sensitivity and glycemic control in older adults [2,84,85,86,87,88]. A review of previously published resistance training studies found that elderly individuals that underwent 3–6 months of moderate-intensity resistance training, could increase whole-body insulin sensitivity by ~10–30% [89]. As expected, improvements in whole-body insulin sensitivity parallel increases in skeletal muscle glucose uptake with resistance training. Bucci et al. [84] demonstrated that 4 months of resistance training could normalize insulin-stimulated glucose uptake in thigh muscle (measured by ^8^F-fluoro-2-deoxyglucose and positron emission tomography), in elderly women born from obese mothers to that of controls. Despite the well-documented increases in lean body mass associated with resistance training, [90,91], increases in insulin sensitivity cannot be accounted for by increases in muscle mass alone [92,93,94], pointing to improvements in skeletal muscle insulin signaling.

### 7.1. Resistance Training and Skeletal Muscle Insulin Signaling in Older Adults

We previously reported that 3 months of moderate-intensity resistance training involving major muscle groups of the upper and lower body, increased insulin sensitivity to a similar extent (~12%) as moderate-intensity endurance training in older men and women [2]. Like the effects of endurance training, we observed enhanced insulin-stimulated AS160 phosphorylation on sites threonine-642 and serine-666 in older adults. However, unlike endurance training, resistance training did not rescue age-related impairments in insulin-stimulated AS160 serine-588 phosphorylation [2]. The regulation of the site-specific phosphorylation of AS160 remains unclear; however, a regression analysis of key demographic variables determined that percent body fat was the strongest independent predictor of insulin-stimulated AS160 serine-588 phosphorylation [2]. Given that body fat declined in response to endurance, but not resistance training, it is possible that exercise training requires a reduction in body fat to reverse age-related impairments in insulin-stimulated AS160 serine-588 phosphorylation. With respect to total protein, we did observe a trend to increase Akt2 protein levels in both young and old adults with resistance training [2]. Similarly, Holton et al. [93] reported that 6 weeks of single-legged strength training increased skeletal muscle insulin receptor and Akt protein levels in non-obese, aged (mean age: 62 years) controls and type 2 diabetic men. Of interest, Akt increases were only observed in the exercised leg indicating that these adaptations are due to local muscle contraction rather than other systematic adaptations [93].

In addition to potential improvements in AS160 phosphorylation, skeletal muscle GLUT4 protein content has been reported to increase with resistance training but may be dependent on pre-training body composition and diabetes status. Six weeks of one-legged strength training (three times per week) in aged, non-obese, type 2 diabetics increased skeletal muscle GLUT4 in the exercised leg (~40%), but not in healthy controls [93]. In support of these findings, 16 weeks of resistance training increased skeletal muscle GLUT4 protein content in middle-aged (30–54 years) prediabetic, obese men [95] and increased the hypomethylation (promotes gene transcription) of the GLUT4 promoter in middle-aged morbidly obese Maori and Pacific Islanders diagnosed with type 2 diabetes [96]. In contrast to these findings, no changes in skeletal muscle GLUT4 were observed in healthy, non-obese, aged individuals in response to 6 weeks of one-legged strength training [93] or 12 weeks of whole body resistance training [2]. In summary, resistance training appears to enhance skeletal muscle insulin signaling at the level of AS160 (and potentially Akt capacity) but may not enhance GLUT4 expression, at least in non-obese, healthy older adults. In contrast, older adults suffering from obesity and/or type 2 diabetes appear to be more sensitive to increases in skeletal muscle GLUT4 protein content, which could contribute to their improved insulin sensitivity with resistance training.

### 7.2. Resistance Training and Glycogen Synthase Pathway in Older Adults

Early research highlighted the beneficial effects of resistance training on nonoxidative glucose disposal in older adults [97]. Miller et al. [94] reported that 16 weeks of resistance training in men between the ages of 50 and 63 increased insulin-stimulated nonoxidative glucose disposal by 40%, likely contributing to the improved whole-body insulin sensitivity (22%) and suggesting resistance training could improve skeletal muscle glycogen metabolism. Despite this finding, the effects of resistance training on the glycogen synthase pathway have been conflicting in older adults. Sixteen weeks of progressive resistance training has been reported to improve glycemic control and increase skeletal muscle glycogen content in older, obese, Latino men and women with type 2 diabetes [86]. Similarly, Holten et al. [93] reported that both healthy and type diabetic older adults had a tendency to increase skeletal muscle glycogen content (~16%) and significantly increased basal glycogen synthase activity (~9% and 20%, respectively) after 6 weeks of resistance training. It is possible that the effects of resistance training on the glycogen synthase pathway are dependent on pre-training glucose tolerance and/or insulin sensitivity given that improvements in glycogen synthase activity in type 2 diabetics were nearly double that of healthy controls [93]. Further, despite similar increases in whole-body insulin sensitivity (~20%–25%) after 6 months of endurance or resistance training, only endurance training improved insulin-stimulated glycogen synthase activity in aged non-diabetic (non-obese) men [66] and we did not observe changes in insulin-stimulated skeletal muscle glycogen synthase dephosphorylation (required for glycogen synthase activity) with resistance training in older, healthy (non-obese) adults [41]. Taken together, these data suggest that resistance training may be especially effective in improving the glycogen synthase pathway in older adults that are also suffering from type 2 diabetes.

### 7.3. Resistance Training and Glucose Oxidation Pathway in Older Adults

Whereas resistance training has produced conflicting results on mitochondrial content and function in young individuals [98,99,100,101], positive effects have been observed in older adults [102,103,104,105]. Jubrias and colleagues [105] reported that despite improved skeletal muscle oxidative capacity in elderly individuals with either endurance or resistance training, mitochondrial content was only increased with resistance training. Improved skeletal muscle mitochondrial area and density have also been reported with 6 months of progressive resistance training in post-menopausal women [104]. Given that resistance training habitually increases mitochondrial content in older adults, but not younger subjects [100,101], this suggests aging muscle may be more sensitive to diverse stimuli to promote mitochondrial content to enhance skeletal muscle oxidative capacity and insulin sensitivity.

Unfortunately, the available research examining the effects of resistance training on insulin-stimulated glucose oxidation in the elderly is sparse, but a few interesting findings have been published. Miller and colleagues [94] did not observe changes in whole-body glucose oxidation in response to 16 weeks of strength training in healthy, older adults. In contrast, skeletal muscle pyruvate oxidation (measured ex vivo) increased in response to 9 months of resistance training in older, obese, type 2 diabetics [106], suggesting resistance training improvements in glucose oxidation may be specific to skeletal muscle. Despite not measuring glucose oxidation, we did measure key metabolic pathways responsible for skeletal muscle glucose oxidation in response to resistance training in young and older adults [41]. Similar to our findings with endurance training, twelve weeks of resistance training decreased insulin-stimulated plasma lactate when normalized to glucose uptake in older adults, suggesting improved shuttling of glucose away from anerobic metabolism [41]. In addition, we reported that older adults had enhanced insulin-stimulated PDH dephosphorylation (responsible for PDH activity) in response to resistance training, which exceeded the response of younger adults [41]. In addition to improvements in PDH regulation, we also reported that resistance training upregulated skeletal muscle MPC1, the transporter responsible for pyruvate entry into the mitochondria [41]. Of interest, these increases were nearly two-fold greater in aged compared to young individuals, [41]. Collectively, these findings suggest that older adults may be especially responsive to mitochondrial adaptations with resistance training, including changes that would permit improved insulin-stimulated skeletal muscle glucose oxidation.

### 7.4. Resistance Training and IMCL in Older Adults

Unlike the effects of endurance training, resistance training appears to have either no effect [76,84] or decreases IMCL [84]. For example, Bucci et al. [84] reported that 4 months of resistance training decreased IMCL in elderly women born from obese mothers, whereas controls had no change in IMCL. Ngo and colleagues [76] provided evidence that fourteen weeks of resistance training had no effect on deltoid IMCL in elderly men (mean age: 72 years) [76]. Of interest, this study [76] also reported that skeletal muscle mitochondrial content increased (elevated citrate synthase) with resistance training; however, skeletal muscle β-hydroxyacyl coenzyme A dehydrogenase (β-HAD), a key enzyme responsible for lipid oxidation, did not change. These findings suggest that for the athlete’s paradox to occur (i.e., IMCL stores increase), exercise training adaptations must include increases in both mitochondrial content and lipid oxidation. The fact that resistance training has a greater glycolytic demand (and lesser lipid oxidation demand) than endurance training likely explains why IMCL stores do not increase with resistance training in older adults. The type of muscle contraction used during exercise may also play a role on IMCL stores. Mueller et al. [107] reported that 12 weeks of eccentric exercise training decreased IMCL in elderly men and women (mean age: 80 years), whereas concentric exercise had no effect. Overall, these findings suggest metabolic demand and the type of muscle contraction during resistance training may play a role in determining whether IMCL stores will decrease or remain unchanged in response to resistance training in older adults.

## 8. Effects of Exercise Training on Skeletal Muscle Tumor Necrosis Factor (TNF)-Alpha in Older Adults

Finally, it should also be noted that the aging process is associated with increased inflammation, which likely contributes to age-related insulin resistance [108]. Skeletal muscle tumor necrosis factor (TNF)-alpha, an inflammatory marker, has been reported to be increased in aged individuals compared to their younger counterparts [10]. A direct link between TNF-alpha and impaired skeletal muscle insulin signaling and glucose uptake has been established in cell culture [109]. In addition, TNF-alpha infusion has been shown to impair skeletal AS160 phosphorylation in healthy individuals [110], providing further evidence this inflammatory protein may contribute to age-related insulin resistance. Interestingly, high-intensity exercise training (cycling and circuit training) [111], moderate-intensity resistance training [10], or a combination of resistance, endurance, and flexibility training [112] have been proven to decrease skeletal muscle TNF-alpha levels in the elderly. Therefore, it is possible that reductions in TNF-alpha or other inflammatory markers could contribute, at least in part, to the exercise-induced improvements in insulin sensitivity in older adults.

## 9. Conclusions

Aging is associated with insulin resistance and increased risk of type 2 diabetes. Age-related structural and metabolic changes to skeletal muscle, including reduced muscle mass, impaired insulin signaling, defects in glucose utilization involving both oxidative and glycogen synthase pathways, likely contribute to this reduced insulin action. In general, endurance training enhances skeletal muscle insulin-stimulated AS160 phosphorylation, GLUT4 protein content and the glycogen synthase and oxidative (PDH) pathways in healthy older adults (Table 1). Similar benefits can be achieved with resistance training in previously healthy older adults, with the exception that enhancements in skeletal muscle GLUT4 and insulin-stimulated glycogen synthase pathways may be more limited (Table 1). When older adults are also classified as obese and/or type 2 diabetic, these individuals may experience greater improvements to the glycogen synthase pathway and improved GLUT4 content with either endurance or resistance training, likely contributing to their improved insulin sensitivity with exercise training.

## Figures and Tables

**Table 1:** Summary of Endurance and Resistance Training Effects on Skeletal Muscle Metabolism in Older Adults.

	Endurance Training	Resistance Training
Whole-Body Insulin Sensitivity	↑	↑
Skeletal Muscle Insulin Signaling		
AS160 Insulin Signaling	↑, ↔ (obesity)	↑
GLUT4	↑	↔, ↑ (obesity)
Glycogen Synthase Activity	↑	↔, ↑ (type 2 diabetic)
PDH Regulation	↑	↑
Skeletal Muscle Mitochondria	↑	↑
Skeletal Muscle IMCL	↑	↔ or ↓
Skeletal Muscle TNF-alpha	↓	↓

PDH, pyruvate dehydrogenase; IMCL, intramyocellular lipid; TNF, tumor necrosis factor.
